# Supplementary material for: Federated hierarchical MARL for zero-shot cyber defense
Source: PLoS One. 2025 Aug 8;20(8):e0329969. doi: 10.1371/journal.pone.0329969 (PMC12334051; doi:10.1371/journal.pone.0329969)
Supplement: Attachment — Submitted filename: Response to Reviewers V3.pdf [file pone.0329969.s003.pdf]

Dear Zeashan Hameed Khan,

I sincerely thank you and the reviewers for the time and effort you devoted to evaluating our manuscript, titled " Federated Hierarchical MARL for Zero-Shot Cyber Defense” ONE-D-25-16867.

I appreciate the thoughtful and constructive comments, which have helped us improve the clarity and quality of our work.

I have carefully considered each point raised and revised the manuscript accordingly. Below, I provide a detailed response to each comment. Journal requirements and additional editor comments are listed below in black, and my responses are provided directly below each point “highlighted in green”

For the grammar check, I have highlighted the text in RED in the modification version.

I hope that the revisions and clarifications meet your expectations and respectfully resubmit our manuscript for your reconsideration.

Journal Requirements:

1. If the reviewer comments include a recommendation to cite specific previously published works, please review and evaluate these publications to determine whether they are relevant and should be cited. There is no requirement to cite these works unless the editor has indicated otherwise.

2. Please review your reference list to ensure that it is complete and correct. If you have cited papers that have been retracted, please include the rationale for doing so in the manuscript text, or remove these references and replace them with relevant current references. Any changes to the reference list should be mentioned in the rebuttal letter that accompanies your revised manuscript. If you need to cite a retracted article, indicate the article’s retracted status in the References list and also include a citation and full reference for the retraction notice.

I have checked each of the 44 references in the manuscript. The majority of the citations are correct and refer to established, non-retracted works. However, I identified a few areas that require complete and accurate referencing. I thank you for pointing out this.

Here are the specific references that need to be updated:

- **Reference: Nguyen TT, Reddi VJ.**
    - **Issue:** The publication year is listed as 2019, but the correct year is 2020.
    - **Correction:** Nguyen TT, Reddi VJ. Deep reinforcement learning for cyber security. IEEE Transactions on Neural Networks and Learning Systems. 2020;31(7):2669-2683.

- **Reference: Nguyen TT, Reddi VJ.**
    - **Issue:** This is a duplicate of reference, all in-text citations ([2] and [11]) and also contains the incorrect year.
    - **Correction:** This reference has been corrected and now the corresponding text also updated and cited as one reference [2]. And also updated to the correct 2020 publication year.
- **This is reference [2] and already updated in the manuscript.**

- **Reference: Alshamrani A, Alshahrani A.**
    - **Issue:** The reference is incomplete and missing the page ranges.
    - **Correction:** Alshamrani A, Alshahrani A. Adaptive Cyber Defense Technique Based on Multiagent Reinforcement Learning Strategies. Intelligent Automation & Soft Computing. 2023;36(3):2539-2555.

Additional Editor Comments:

The paper has been improved technically but the write up must be polished by a technical English expert for clarity.

I have asked an expert to help on checking and revising my article and based on her recommendation, I have changed the manuscript and highlighted the changes in RED and also some words are removed but for easily tracking them, I have used Strikethrough text in the file “version 3 with modifications”. However, the clean version is also provided.

There is one point I would like to mention here, the English expert asked me to change “I” where possible to “we”, however, I proceed using the first-person singular ("I") as directed by one the reviewer in the previous revision of the article.

Thanks

Adel Alshamrani, PhD
