# Supplementary material for: Federated hierarchical MARL for zero-shot cyber defense
Source: PLoS One. 2025 Aug 8;20(8):e0329969. doi: 10.1371/journal.pone.0329969 (PMC12334051; doi:10.1371/journal.pone.0329969)
Supplement: Attachment — Submitted filename: Response to Reviewers.docx [file pone.0329969.s001.docx]

Dear Zeashan Hameed Khan,

We sincerely thank you and the reviewers for the time and effort you devoted to evaluating our manuscript, titled " Federated Hierarchical MARL for Zero-Shot Cyber Defense” ONE-D-25-16867.

We appreciate the thoughtful and constructive comments, which have helped us improve the clarity and quality of our work.

We have carefully considered each point raised and revised the manuscript accordingly. Below, we provide a detailed response to each comment. Reviewer comments are listed in black, and our responses are provided directly below each point in green, yellow, and blue.

We hope that the revisions and clarifications meet your expectations and respectfully resubmit our manuscript for your reconsideration.

Journal Requirements:

When submitting your revision, we need you to address these additional requirements.

1. Please ensure that your manuscript meets PLOS ONE's style requirements, including those for file naming. The PLOS ONE style templates can be found at

https://journals.plos.org/plosone/s/file?id=wjVg/PLOSOne_formatting_sample_main_body.pdf and

[https://journals.plos.org/plosone/s/file?id=ba62/PLOSOne_formatting_sample_title_authors_affiliations.pdf](https://eur02.safelinks.protection.outlook.com/?url=https%3A%2F%2Ftrack.editorialmanager.com%2FCL0%2Fhttps%3A%252F%252Fjournals.plos.org%252Fplosone%252Fs%252Ffile%253Fid%3Dba62%252FPLOSOne_formatting_sample_title_authors_affiliations.pdf%2F1%2F010f01969f909524-205fdb62-e3de-4090-8682-3494db0e9a17-000000%2FwXf7FAluF9eNGwCUaRFNIRtIURVJlawc7mGc8k1AWfY%3D209&data=05%7C02%7Casalshamrani%40uj.edu.sa%7C741d9570d1274fb92c7b08dd8baef846%7Cb453d91b6ac14b61b8b85e65e422233f%7C0%7C0%7C638820306078379061%7CUnknown%7CTWFpbGZsb3d8eyJFbXB0eU1hcGkiOnRydWUsIlYiOiIwLjAuMDAwMCIsIlAiOiJXaW4zMiIsIkFOIjoiTWFpbCIsIldUIjoyfQ%3D%3D%7C0%7C%7C%7C&sdata=jM2a75v99cwfE2vxz7scaR0nJ00felMSjUyG1QscX2k%3D&reserved=0)

( Doubled checked and followed

2. Please note that PLOS ONE has specific guidelines on code sharing for submissions in which author-generated code underpins the findings in the manuscript. In these cases, we expect all author-generated code to be made available without restrictions upon publication of the work. Please review our guidelines at https://journals.plos.org/plosone/s/materials-and-software-sharing#loc-sharing-code and ensure that your code is shared in a way that follows best practice and facilitates reproducibility and reuse.

3. We note that your Data Availability Statement is currently as follows: All relevant data are within the manuscript and its Supporting Information files.

Please confirm at this time whether or not your submission contains all raw data required to replicate the results of your study. Authors must share the “minimal data set” for their submission. PLOS defines the minimal data set to consist of the data required to replicate all study findings reported in the article, as well as related metadata and methods (https://journals.plos.org/plosone/s/data-availability#loc-minimal-data-set-definition).

For example, authors should submit the following data:

- The values behind the means, standard deviations and other measures reported;
- The values used to build graphs;
- The points extracted from images for analysis.

Authors do not need to submit their entire data set if only a portion of the data was used in the reported study.

If your submission does not contain these data, please either upload them as Supporting Information files or deposit them to a stable, public repository and provide us with the relevant URLs, DOIs, or accession numbers. For a list of recommended repositories, please see https://journals.plos.org/plosone/s/recommended-repositories.

If there are ethical or legal restrictions on sharing a de-identified data set, please explain them in detail (e.g., data contain potentially sensitive information, data are owned by a third-party organization, etc.) and who has imposed them (e.g., an ethics committee). Please also provide contact information for a data access committee, ethics committee, or other institutional body to which data requests may be sent. If data are owned by a third party, please indicate how others may request data access.

4. When completing the data availability statement of the submission form, you indicated that you will make your data available on acceptance. We strongly recommend all authors decide on a data sharing plan before acceptance, as the process can be lengthy and hold up publication timelines. Please note that, though access restrictions are acceptable now, your entire data will need to be made freely accessible if your manuscript is accepted for publication. This policy applies to all data except where public deposition would breach compliance with the protocol approved by your research ethics board. If you are unable to adhere to our open data policy, please kindly revise your statement to explain your reasoning and we will seek the editor's input on an exemption. Please be assured that, once you have provided your new statement, the assessment of your exemption will not hold up the peer review process.

**Additional Editor Comments:**

The paper proposes an interesting solution to the cyber security of the networked devices. However, it needs significant improvement before further consideration.
The MARL algorithm needs more concrete references and the link to datasets must be included in the references and acknowledgement.
Please take a careful look at the reviewer's comments and submit a revised version by the due date.

This study is based on data derived from the DARPA Cyber Range and Test Environment (CRATE) dataset (2022). The dataset is not publicly available, and details about its access and distribution are limited. All experiments and analyses comply with applicable institutional and ethical guidelines.

[Note: HTML markup is below. Please do not edit.]

Reviewers' comments:

5. Review Comments to the Author

Please use the space provided to explain your answers to the questions above. You may also include additional comments for the author, including concerns about dual publication, research ethics, or publication ethics. (Please upload your review as an attachment if it exceeds 20,000 characters)

Reviewer #1: Please find below some comments that may help to improve the quality of this manuscript.

Author summary: The "Author summary" section contains placeholder "Lorem ipsum" text and needs to be written.

I removed this from the template.

1.4 Evaluation: The authors should add a detailed description of the network topology and how many agents were deployed in the network. The goal is to share enough information so independent researchers can reproduce the results discussed in the manuscript (say in an Evaluation Setup section). Moreover, remember that the evaluation setup is important for the interpretation of quantitative results of a model combining hierarchical MARL and Federated Knowledge Sharing.

I have added shorter version of this response to the manuscript in Evaluation section page 16 and 17. I will add the full response if needed.

To rigorously evaluate the proposed Adaptive Zero-Shot Hierarchical MARL (AZH-MARL) framework, we designed a comprehensive experimental setup utilizing both established simulation environments and a custom-developed testbed. This section details the network topologies, agent configurations, and evaluation scenarios employed.

**Evaluation Environments:**

Our experiments were conducted across three primary environments to assess different facets of the AZH-MARL framework:

1. **CybORG Simulation Environment:** We utilized the CybORG environment [39], a recognized platform for cybersecurity research developed for the CAGE Challenge. For our experiments, we configured a simulated enterprise network topology within CybORG consisting of approximately 50 nodes distributed across three distinct subnets: a public-facing DMZ, an internal corporate network, and a restricted operational technology (OT) segment. These segments included a variety of simulated devices such as web servers, database servers, user workstations, and programmable logic controllers (PLCs). The connectivity was designed to mimic realistic enterprise traffic flows and potential attack paths. Attack scenarios native to CybORG, as well as custom scripted attacks, were used to test the defensive capabilities of our agents in this dynamic setting.
2. **DARPA CRATE Dataset Scenarios:** The DARPA Cyber Range and Test Environment (CRATE) Dataset [40] provided a rich source of realistic network traffic and attack data. While the full dataset is extensive, we selected specific scenarios relevant to testing zero-shot learning capabilities. These scenarios involved diverse attack vectors and sophisticated adversarial behaviors. The data from CRATE was primarily used to train and evaluate the semantic mapping function and the zero-shot policy transfer mechanisms of the AZH-MARL framework, providing a basis for assessing performance against previously unseen attack patterns.

However, This study is based on data derived from the DARPA Cyber Range and Test Environment (CRATE) dataset (2022). The dataset is not publicly available, and details about its access and distribution are limited. All experiments and analyses comply with applicable institutional and ethical guidelines.

3. **Custom Federated Learning Testbed:** To specifically evaluate the federated learning and privacy-preserving aspects of AZH-MARL, we developed a custom distributed testbed. This testbed simulated a collaborative defense scenario involving three independent organizational networks. Each virtual network environment within the testbed comprised approximately 30-40 nodes, with unique security policies, varying levels of baseline security posture, and distinct, locally generated attack patterns (e.g., phishing campaigns targeting one network, ransomware in another, data exfiltration attempts in the third). This heterogeneity was crucial for testing the framework’s ability to share knowledge effectively and adapt defenses in a federated manner while preserving the privacy of each participating entity’s local data.

**Agent Deployment and Configuration:**

In each environment where applicable (CybORG and the Custom Federated Learning Testbed), the AZH-MARL framework was deployed with a hierarchical agent structure:

- **Meta-Controller:** A single meta-controller agent was responsible for overseeing the overall defense strategy within each simulated network (or each federated node in the custom testbed). It received aggregated security metrics and threat assessments as its state input and selected high-level sub-policies (options) for execution.
- **Sub-Level Agents:** Four types of specialized sub-level agents were deployed, corresponding to the primary defense tasks:
  - **Reconnaissance Agent:** Monitored network traffic, system logs, and endpoint behaviors to detect suspicious activities and potential threats.
  - **Analysis Agent:** Evaluated detected anomalies and alerts to determine their nature, severity, and potential impact, correlating information from various sources.
  - **Response Agent:** Executed defensive actions, such as isolating compromised hosts, blocking malicious traffic, or deploying countermeasures, based on the analysis and meta-controller directives.
  - **Recovery Agent:** Focused on restoring affected systems and services to normal operation post-incident, including patching vulnerabilities and verifying system integrity.

In the CybORG environment, these agents operated on the simulated enterprise network. In the custom federated learning testbed, each of the three simulated organizational networks had its own deployment of this hierarchical agent structure. The local models from these agents were then aggregated via the federated learning mechanism described in Section 1.1.

The number of primitive actions available to sub-level agents varied based on the task and environment but typically ranged from 5 to 10 distinct actions per agent type (e.g., for a response agent: block IP, isolate host, patch vulnerability, etc.). The meta-controller selected from the four sub-level policy types. All agents were trained using Proximal Policy Optimization (PPO) as detailed in Section 0.6.3.

This detailed experimental setup was designed to provide a robust and reproducible evaluation of the AZH-MARL framework’s capabilities in complex and evolving cyber threat landscapes

1.4.3 Figure 2 (Detection Performance) seems to mislabel some approaches compared to Table 1. For example, Figure 2 shows "Federated Learning" and "Zero-Shot Learning" as separate defense approaches, whereas Table 1 lists "Centralized Learning" and "Independent Learning". This inconsistency needs correction.

Fixed in the manuscript and now it appears as Figure 3 since I added a new Figure to the manuscript.

1.4.3 Results and Analysis: The authors should explain how "zero-day exploits" and "advanced persistent threats" were defined and executed during the experiments.

Here I added the full response to the respected author, but I have provided shorter response in the manuscript for the seek of keeping it easily understandable, but if you prefer to add the full response to the manuscript then I will

**Zero-Day Exploits**

Definition:

In the context of our experiments, a "zero-day exploit" refers to an attack that leverages a previously unknown vulnerability in the target systems or software, for which no specific signature or patch was available at the time of the simulated attack. The key characteristic is the novelty of the attack vector to the defense system being evaluated. Our AZH-MARL framework, with its zero-shot learning component, is specifically designed to address such threats by generalizing from known attack patterns to recognize and respond to these novel instances.

**Execution and Simulation:**

To simulate zero-day exploits, we employed the following methodologies across our evaluation environments:

1. CybORG Simulation Environment: We utilized attack scenarios within CybORG that were either newly developed or modified versions of existing attacks, ensuring that the specific attack signatures or behavioral patterns were not part of the training data for the baseline defense agents or the initial training phase of our AZH-MARL agents. This was achieved by holding out specific attack types or variants from the training set and introducing them only during the evaluation phase.

2. DARPA Dataset Scenarios: The dataset contains a diverse range of attack instances. For zero-shot evaluation, we partitioned the dataset such that attack classes or specific instances with unique characteristics (e.g., novel combinations of tactics, techniques, and procedures - TTPs) were reserved for the test set. The agents were trained on a distinct set of known attack types, and their ability to detect and respond to these unseen types from the test set constituted the zero-day exploit scenario.

3. Custom Federated Learning Testbed: Within each simulated organizational network in our custom testbed, we introduced novel attack scripts or modified existing attack tools to generate traffic and host behaviors that were not previously encountered by the local agents or the federated model. This involved, for example, using obfuscated versions of known malware, new phishing campaign templates, or exploiting simulated vulnerabilities that were not present in the training environments of other federated nodes.

The core principle was to ensure that the defense system had no prior explicit knowledge (e.g., signatures, specific rules) of the exact exploit being used during these test scenarios, forcing it to rely on generalized threat understanding and adaptive capabilities.

**Advanced Persistent Threats (APTs)**

Definition:

An "Advanced Persistent Threat (APT)" in our experimental context is defined as a sophisticated, multi-stage cyber attack campaign characterized by:

- Stealth and Evasion: APT actors employ techniques to avoid detection by standard security measures.
- Persistence: Attackers aim to maintain long-term access to the target network.
- Targeted Objectives: APTs are typically goal-oriented, often focused on espionage, data exfiltration, or strategic disruption.
- Multiple Stages: The campaign involves several phases, such as initial reconnaissance, gaining entry, lateral movement, privilege escalation, establishing command and control (C2), and achieving the final objective.

**Execution and Simulation:**

Simulating APTs requires modeling these complex, long-duration characteristics:

1. CybORG Simulation Environment: We leveraged CybORG’s capabilities to script multi-stage attack scenarios that mimic APT behavior. This involved chaining multiple attack actions over time, where the success of one stage enabled the next. For example, an initial phishing attack (reconnaissance/entry) might be followed by malware deployment (persistence), lateral movement to access sensitive servers, and finally, data exfiltration attempts. The defense agents were evaluated on their ability to detect and disrupt these linked activities across the different stages of the simulated APT campaign.
2. DARPA Dataset Scenarios: While the dataset primarily consists of captured network traffic and logs, certain scenarios within it exhibit characteristics consistent with APTs, such as prolonged low-and-slow attacks or coordinated multi-vector intrusions. We selected and analyzed these scenarios to evaluate the AZH-MARL framework’s ability to piece together disparate, subtle indicators that might signify an ongoing APT.
3. Custom Federated Learning Testbed: We designed specific APT-like scenarios within our custom testbed. For instance, one federated node might experience a slow reconnaissance phase, followed by a targeted spear-phishing attack leading to a compromised host. This host would then attempt to communicate with an external C2 server and perform internal network scanning (lateral movement preparation). The evaluation focused on the ability of the local and federated system to detect these coordinated, low-observable activities over an extended period.

The evaluation of defenses against APTs focused not just on detecting individual malicious actions but also on recognizing the broader campaign and responding in a coordinated manner to disrupt the attacker’s overall objectives. The hierarchical nature of AZH-MARL was particularly relevant here, with the meta-controller aiming to understand the high-level threat context indicative of an APT.

The added shorten response to the manuscript :

\paragraph{Zero-Day Exploits.}

In our experiments, zero-day exploits refer to attacks leveraging previously unknown vulnerabilities for which no explicit signature or patch was available. These were simulated across CybORG, the DARPA dataset, and our custom federated testbed by withholding specific attack variants from training. This forced agents—especially those using our AZH-MARL framework with zero-shot learning—to generalize from known behaviors and respond adaptively to novel threats, such as obfuscated malware or unseen TTPs.

\paragraph{Advanced Persistent Threats (APTs).}

APTs were modeled as multi-stage, stealthy campaigns with persistent access goals. Using CybORG and selected DARPA scenarios, we scripted chained attack phases (e.g., phishing, lateral movement, data exfiltration), testing agents' ability to detect the campaign across time. In our federated testbed, APTs unfolded gradually within different nodes, evaluating the meta-controller’s capacity to recognize strategic threats and orchestrate coordinated responses beyond isolated alerts.

Dataset: Please add a link to the DARPA CRATE dataset used in this investigation.

Will check

Reviewer #2: This work proposes an adaptive zero-shot hierarchical multiagent reinforcement learning approach to achieve efficient and resilient cyber defense systems. The result shows that the proposed framework is also able to accurately detect zero-day with a relatively lower response time.

Although the paper uses real-testbed evaluations however some implementation details need more explanation (/references). Also, the problem and proposed mechanism needs proper justification.

1. All equations and symbols used must be explained clearly in the paper. Eq. 1 is misplaced and is never referenced in the literature which makes it hard to follow.
The contrastive loss function Equation in section 1.3.5 needed to be properly written and labelled. Use a table to explain each equation symbol in use.

Fixed in the manuscript and also I added a new table, in the appendix section, to explain the symbols used in all equations

Part of the appendix is screenshotted here:

2. Also please use a table to list all abbreviations. Some statements are confusing, for example the second line of second last paragraph of section 0.1 says, "The work likely addresses the challenge of scalability and coordination in
multi-agent systems ..." Please rewrite it.

Added new section in the manuscript List of Abbreviations after the reference section

3. Algorithm 1 (Adaptive Zero Shot Learning algorithm) needs better explanation (e.g. components running it?) along with the reference in the text.

**Explanation:**
Algorithm 1 is executed by each **local defender agent** during online inference. The key components involved are:

- **Semantic Mapper (ϕ):** Projects observed features into the learned semantic space.
- **Policy Matching Module:** Compares the mapped vector to existing known responses using similarity functions.
- **Knowledge Base:** A local copy or federated view of known defense-action mappings.
- **Adaptation Module:** If no match is above threshold, the agent uses fallback exploration or queries the federation for new mappings.

The algorithm runs continuously on agents deployed in edge hosts or gateways, depending on the testbed scenario. The logic is integrated as part of the agent's decision cycle.

I am also adding this table for more clarification, but not sure if should we keep it in the paper or not since I believe these are extra information. However, I can remove it to from the paper if the respected reviewers suggest that.

| **Text Description** | **Algorithm Elements** |
| --- | --- |
| **Semantic Mapper (ϕ)** | Line 5: zₜ = ϕ(xₜ) — computes semantic embedding from observed attack pattern. |
| **Policy Matching Module** | Line 6: if similarity(zₜ, Z_known) < threshold — similarity check to known responses, used to classify as novel or known attack. |
| **Knowledge Base** | Z_known is updated in Line 12 and used in Line 6 for comparison — effectively a knowledge base of embeddings. |
| **Adaptation Module** | Line 7–13: Handles zero-shot detection and policy-based action for novel attacks. Also includes fallback (zero-shot transfer) when unfamiliar attacks arise. |
| **Online execution at edge agents** | Line 2: while not converged do — denotes continuous execution; Line 1 sets up local state (B is the local replay buffer). |
| **Learning updates** | Line 20–24: Policy and semantic mapping updates from sampled buffer experience. |

4. In implementation section please add a table stating all python libraries/tools used, their purpose, and reference.

**Table X: Python Libraries and Tools Used in Implementation (Confirmed from Manuscript)**

The following Python libraries and tools are explicitly mentioned in the implementation:

| **Library/Tool** | **Purpose** | **Reference/Version (as per manuscript)** |
| --- | --- | --- |
| Python | Core programming language for framework development and experimentation. | 3.8 (mentioned in Section 1.3) |
| PyTorch | Deep learning framework used for implementing the neural network models. | 1.9 (mentioned in Section 1.3) |
| Ray RLlib | Reinforcement learning library used for training the MARL agents. | Version not specified in manuscript. (Consider adding official website: <https://ray.io/rllib> or the specific version used if known) |
| CybORG | Cyber security simulation environment used for simulating enterprise network scenarios and evaluating defense agents. | [39] (referenced in Section 1.3 and throughout) |

5. Proper references should be provided with baseline approaches listed in section 1.4.2.

I have added the references to each approach and also added this to the manuscript.

**Summaries of Baseline Approaches:**

1. **Traditional MARL**: A flat multi-agent reinforcement learning model without

hierarchy or transfer mechanisms [9, 11];

2. **Hierarchical MARL**: A structured MARL approach that incorporates

hierarchical control but lacks generalization capabilities [16–19];

3. **Federated Learning**: A collaborative training method where agents share

models without centralizing raw data [25, 26, 28, 29];

4. **Zero-Shot Learning**: A generalization-based model trained to respond to unseen attack types without prior specific training [20–24].

6. Implementation and results sections require thorough revisions, especially specific details regarding implementation and evaluation needs to be added in the corresponding sections.
For example, the custom federated learning testbed used for performance evaluation should either be explained clearly (with figure) or a reference needs to be added.

To specifically evaluate the federated learning and privacy-preserving aspects of AZH-MARL, we developed a custom distributed testbed. This testbed simulated a collaborative defense scenario involving three independent organizational networks. Each virtual network environment within the testbed comprised approximately 30-40 nodes, with unique security policies, varying levels of baseline security posture, and distinct, locally generated attack patterns (e.g., phishing campaigns targeting one network, ransomware in another, data exfiltration attempts in the third). This heterogeneity was crucial for testing the framework’s ability to share knowledge effectively and adapt defenses in a federated manner while preserving the privacy of each participating entity’s local data.

In the custom federated learning testbed, each of the three simulated organizational networks had its own deployment of this hierarchical agent structure (Meta-Controller and four sub-level agents: Reconnaissance, Analysis, Response, Recovery). The local models from these agents were then aggregated via the federated learning mechanism.

7. From the results in Fig 2, it looks like the combination of Federated and zero-shot learning (if added) may result in an overall better or similar performance to that of the proposed (AZH-MARL) one. Please elaborate on it.

While both federated learning and zero-shot learning independently offer strong performance in terms of generalization and decentralized training, they each have limitations when applied in isolation. Federated learning alone suffers from difficulty in adapting to unseen attack patterns, particularly those not present in the training data across agents. Zero-shot learning, while effective in detecting novel attacks, often lacks context-aware response strategies and is susceptible to noisy or ambiguous semantic mappings when not anchored to local experience.

The proposed AZH-MARL framework builds on the strengths of both techniques: it uses federated knowledge sharing to continuously refine distributed policies and semantic mappings, while leveraging a hierarchical multi-agent architecture to locally adapt to emergent threats. This tight coupling of ZSL within a federated MARL framework enables both **faster response times** and **higher detection robustness**, especially in the presence of adversarial behavior or concept drift. As shown in Figure 2, AZH-MARL outperforms either technique in isolation by providing **adaptive coordination** between semantic embedding and policy optimization across distributed agents.

While Federated Learning (FL) and Zero-Shot Learning (ZSL) together **offer strong generalization**, **they lack adaptive coordination and localized response intelligence**, which AZH-MARL provides.
