# Supplementary material for: Federated hierarchical MARL for zero-shot cyber defense
Source: PLoS One. 2025 Aug 8;20(8):e0329969. doi: 10.1371/journal.pone.0329969 (PMC12334051; doi:10.1371/journal.pone.0329969)
Supplement: Attachment — Submitted filename: Response to Reviewers V2.docx [file pone.0329969.s002.docx]

Dear Zeashan Hameed Khan,

I sincerely thank you and the reviewers for the time and effort you devoted to evaluating our manuscript, titled " Federated Hierarchical MARL for Zero-Shot Cyber Defense” ONE-D-25-16867.

I appreciate the thoughtful and constructive comments, which have helped us improve the clarity and quality of our work.

I have carefully considered each point raised and revised the manuscript accordingly. Below, I provide a detailed response to each comment. Reviewer comments are listed in black, and my responses are provided directly below each point.

I hope that the revisions and clarifications meet your expectations and respectfully resubmit our manuscript for your reconsideration.

Review Comments to the Author

Please use the space provided to explain your answers to the questions above. You may also include additional comments for the author, including concerns about dual publication, research ethics, or publication ethics. (Please upload your review as an attachment if it exceeds 20,000 characters)

Reviewer #2: The authors have incorporated my previous comments in this revision. There are a few more comments to add.
1. The fontsize of text shown in tables (3,4,5,6) and figures (4 & 5) should be increased to make them easily readable.
2. Some reference to other relevant works that combines federated and zero-shot learning should also be included.

Reviewer #3: The paper is already revised and the response is acceptable, minor points to address before acceptance please:

1. I have noticed that the paper has one author but the pronouns are all "we" and such, was the manuscript written by a group or a sole author? Please adjust this for the entire paper.
2. The abstract could benefit more from numerical results.

=================	========================	====================

1. The fontsize of text shown in tables (3,4,5,6) and figures (4 & 5) should be increased to make them easily readable.
   Yes, I have changed the size of the font in all tables and figures, hopefully they are now more readable.
2. Some reference to other relevant works that combines federated and zero-shot learning should also be included.

I have added another four recent works as suggested. They are incorporated in the manuscript in section 2.4

\textbf{\textcolor{blue}{Zhou et al. \cite{yang2023adaptive}}} introduced an adaptive federated few-shot learning framework with prototype rectification, demonstrating how semantic prototypes can be aligned across decentralized clients to improve generalization. Though focused on few-shot settings, their prototype correction mechanism offers valuable insights for federated zero-shot adaptation, particularly in scenarios where semantic drift and heterogeneous data distributions hinder model transfer.

\textbf{\textcolor{blue}{Zhao et al. \cite{zhao2022semi}}} introduce a semi‑supervised federated intrusion detection scheme that applies knowledge distillation and voting to cope with non‑IID data and communication constraints. Similarly, the FedGKD approach \textbf{\textcolor{blue}{\cite{zhang2022fedzkt} uses}} global knowledge distillation to address heterogeneity across edge clients in IoT settings. These show how KD can enhance privacy‑preserving and collaborative model learning, and our work extends these ideas by integrating zero or few‑shot generalization within a hierarchical MARL framework

\textbf{\textcolor{blue}{More recently, Wang et al.}} \cite{10.1007/978-3-031-96235-6_24}presented a federated zero‑shot learning framework that uses an LLM to generate privacy-conscious semantic embeddings for unknown attack types. These embeddings are collaboratively shared between clients, enabling zero-day attack detection without exposing raw data. Their approach directly parallels our semantic mapping module and further supports our hierarchical MARL design by validating the feasibility of federated semantic transfer for unseen threats.

==================	==========================	==============

Reviewer #3: The paper is already revised and the response is acceptable, minor points to address before acceptance please:

1. I have noticed that the paper has one author but the pronouns are all "we" and such, was the manuscript written by a group or a sole author? Please adjust this for the entire paper.

I agree to use I instead of we, but I used to use we in academic writing as this is more formal. However, I change it based on you recommendation.

2. The abstract could benefit more from numerical results.

The last part of the abstract has been modified to show and benefit more from the numerical results

The detailed evaluation demonstrates that our approach significantly outperforms existing methods across a range of scenarios. It achieves a high detection rate of 94.2\% for known attacks and 82.7\% for zero-day exploits, while maintaining a low false positive rate of 3.8\%. This robust performance extends to the most sophisticated threats, achieving an 87.3\% containment rate against Advanced Persistent Threats (APTs). The framework's zero-shot capability is underpinned by a semantic mapping accuracy of 89.3\%, which enables rapid adaptation to novel threats. Consequently, the mean response time is reduced by 35\% for known attacks and 42\% for zero-day exploits compared to the best-performing baseline. Furthermore, the federated learning architecture proves highly efficient, reducing communication overhead by 45\% while preserving privacy. These results collectively demonstrate our framework's potential to deliver a new standard of resilient and adaptive cyber defense in complex, distributed environments
